# A randomized study of contingency management and spirometric lung age for motivating smoking cessation among injection drug users

**DOI:** 10.1186/1471-2458-14-761

**Published:** 2014-07-28

**Authors:** Michael B Drummond, Jacquie Astemborski, Allison A Lambert, Scott Goldberg, Maxine L Stitzer, Christian A Merlo, Cynthia S Rand, Robert A Wise, Gregory D Kirk

**Affiliations:** Department of Medicine, School of Medicine, Johns Hopkins University, Baltimore, MD USA; Department of Epidemiology, Bloomberg School of Public Health, Johns Hopkins University, Baltimore, MD USA; University of Chicago School of Medicine, University of Chicago, Chicago, IL USA

**Keywords:** Contingency management, Spirometry, Lung age, Smoking cessation

## Abstract

**Background:**

Even after quitting illicit drugs, tobacco abuse remains a major cause of morbidity and mortality in former injection drug users. An important unmet need in this population is to have effective interventions that can be used in the context of community based care. Contingency management, where a patient receives a monetary incentive for healthy behavior choices, and incorporation of individual counseling regarding spirometric “lung age” (the age of an average healthy individual with similar spirometry) have been shown to improve cessation rates in some populations. The efficacy of these interventions on improving smoking cessation rates has not been studied among current and former injection drug users.

**Methods:**

In a randomized, factorial design study, we recruited 100 active smokers from an ongoing cohort study of current and former injection drug users to assess the impact of contingency management and spirometric lung age on smoking cessation. The primary outcome was 6-month biologically-confirmed smoking cessation comparing contingency management, spirometric lung age or both to usual care. Secondary outcomes included differences in self-reported and biologically-confirmed cessation at interim visits, number of visits attended and quit attempts, smoking rates at interim visits, and changes in Fagerstrom score and self-efficacy.

**Results:**

Six-month biologically-confirmed smoking cessations rates were 4% usual care, 0% lung age, 14% contingency management and 0% for combined lung age and contingency management (p = 0.13). There were no differences in secondary endpoints comparing the four interventions or when pooling the lung age groups. Comparing contingency management to non-contingency management, 6-month cessation rates were not different (7% vs. 2%; p = 0.36), but total number of visits with exhaled carbon monoxide-confirmed abstinence were higher for contingency management than non-contingency management participants (0.38 vs. 0.06; p = 0.03), and more contingency management participants showed reduction in their Fagerstrom score from baseline to follow-up (39% vs. 18%; p = 0.03).

**Conclusions:**

While lung age appeared ineffective, contingency management was associated with more short-term abstinence and lowered nicotine addiction. Contingency management may be a useful tool in development of effective tobacco cessation strategies among current and former injection drug users.

**Trial registration:**

Clinicaltrials.gov NCT01334736 (April 12, 2011).

## Background

Injection drug users (IDUs) have among the highest prevalence of tobacco dependence, yet represent an understudied population in terms of smoking cessation strategies [1–4]. IDUs report smoking prevalence four times that of the general US population [5]. Low socioeconomic status, low educational achievement and polysubstance abuse are prevalent among IDUs and each is independently associated with increased tobacco dependence [6–8]. At the individual level, factors in each of the five psychosocial domains (personality, drug use behavior, family, peer and environment) have been shown to be independently associated with nicotine dependence in an urban cohort [9, 10].

Studies have consistently demonstrated that the majority of illicit drug users are interested in quitting smoking [3, 11]. As many as 61% of IDUs in substance abuse treatment programs report a desire to quit tobacco use, with an average of five prior quit attempts per person [12, 13]. Designing effective and feasible smoking cessation programs tailored for this population may substantially improve cessation. Contingency management is an approach which provides a structured incentive contingent upon changes in a participant’s behavior [14, 15]. Typically, these incentives are in the form of a voucher or monetary reward for achieving a pre-specified therapeutic target. Initially employed as a motivation for illicit drug use cessation [16–19], several studies have demonstrated moderate efficacy in improving tobacco cessation rates [20–24]. A second novel approach to smoking cessation uses the concept of “lung age” as a motivational tool for smoking cessation [25]. Spirometric measurements of lung function are typically reported in absolute terms or percentage of predicted values based upon a referent population. Lung age reports spirometry results using the age of an average healthy individual with similar spirometry results (i.e., “You are 50 years old, but you have the lungs of a 70 year old”). The use of lung age as a motivational tool has been shown to improve biologically-confirmed smoking cessation endpoints in community-based clinic populations [25] and perceived smoking-related risks, worries and desire to quit in college smokers [26]. To date, no study has evaluated the efficacy of the positive reinforcement associated with contingency management and negative reinforcement of lung age-based counseling for improving smoking cessation among IDUs.

The AIDS Linked to the Intravenous Experience (ALIVE) study, a prospective, longitudinal cohort of persons with a history of injecting drugs followed in Baltimore, Maryland since 1988, represents an ideal population to explore novel smoking cessation interventions [27]. This cohort has nearly ubiquitous cigarette smoking [28, 29] and recently instituted serial spirometric measures into the existing data collection protocol. In this study, we assess the impact of contingency management and spirometric lung age as motivational tools to improve 6-month biologically confirmed smoking cessation rates in a cohort of 100 current smokers in a randomized, factorial design study. We hypothesized that the individuals who receive smoking cessation counseling including contingency management or spirometric lung age or both would be more likely to achieve tobacco cessation and have greater change in self-efficacy and intention to quit at 6 months compared to usual care.

## Methods

### Participant recruitment and eligibility

As described previously [27, 30], ALIVE has recruited residents of Baltimore, MD since 1988 who were ≥18 years of age and had a history of injecting drugs. Biannual study visits include standardized questionnaires, a clinical examination, and biospecimen collection. Since 2007, pre-bronchodilator spirometry testing has been performed at each study visit. From March 16, 2011 to February 3, 2012, ALIVE participants presenting for a scheduled lung sub-study visit were screened for inclusion in this trial. Eligibility requirements included current cigarette smoking (defined as a history of smoking at least 100 cigarettes in their lifetime as well as reporting any cigarette smoking in the last month), no current involvement in a smoking cessation program, no current use of nicotine replacement therapy or other smoking cessation pharmacological treatments (bupropion, varenicline), interested in involvement in a smoking cessation trial and the ability to perform spirometry. After screening, the study was described to participants, and if interested, written informed consent was obtained. The study was approved by the IRB of Johns Hopkins University.

### Randomization and study design

Prior to study initiation, 120 sequentially numbered opaque sealed envelopes were externally prepared that included random assignment to one of four interventions. Randomization sequence was computer-generated using a block randomization approach with randomly ordered four and eight sample blocks. After informed consent was obtained and baseline data including smoking-related questionnaires, assessment of self-efficacy and intention to quit smoking, exhaled carbon monoxide (eCO) level and spirometry were completed, study staff was provided the next sequential envelope which assigned the intervention.

### Study visit protocol

The overall design of the study included one baseline visit and six follow-up visits over six months. All visits occurred at the ALIVE research clinic site. Assessment of eligibility, informed consent, randomization and spirometry measurement occurred at the baseline visit. Follow-up visits occurred at one, two and four weeks, then two, three and six months from baseline visit. An increased frequency of visits early in the study was included to enhance protocol compliance.

### Study procedures

At baseline and follow-up visits, all participants completed smoking-related questionnaires, assessment of self-efficacy and intention to quit smoking and eCO measurement. At all visits, participants also received a brief standardized counseling session on the harms of smoking and were offered information on smoking cessation resources available in the community. If the participant inquired about nicotine replacement therapy, he or she was advised to contact their primary care provider to discuss potential therapeutic options. Blood was obtained for serum cotinine analysis at six month visits.

#### Usual care

At baseline and follow-up visits, the usual care group reviewed baseline spirometry results of their lung function reported as a percentage of predicted values, communicated in a standardized written format. Lung function was first described as the numerical percent predicted value, and defined as normal if the forced expiratory volume in one second (FEV1) was equal or greater to 80% of predicted value. If normal, the report stated “Even though your lung function is normal, it is still important to quit smoking to prevent future damage to the lungs.” If the results were abnormal, the report stated “This suggests that your lungs may already be damaged by smoking. Quitting smoking now can slow the rate of damage of the lungs.”

#### Contingency management

Individuals randomized to contingency management received similar interventions as the usual care group. Additionally, it was explained to participants that they would receive monetary compensation for biological confirmation of tobacco cessation. At each visit, exhaled carbon monoxide levels were checked, and if eCO was <7 ppm, participants received compensation in a manner modified from the methodology of Shoptaw [31]. The first negative eCO resulted in $25 payment, with subsequent negative eCO visits increasing $5 in payment to a maximum of $50. If the participant had an eCO consistent with recent tobacco use, they received no payment and the payment structure reset to the starting amount.

#### Lung age intervention

For individuals randomized to lung age intervention, spirometric results were reviewed in the context of lung age [25]. Visual graphs were used to explain how the lung function normally reduces with age and that smoking can damage lung in a manner similar to more rapid aging. The written report included their chronological age and lung age. A similar description of normal or abnormal results was provided as in the usual care intervention, although the threshold to define abnormal was lung age exceeding chronological age.

#### Combined contingency management and lung age

Individuals randomized to this intervention received a combination of the lung age and contingency management protocols described above.

### Study measures

Pre-bronchodilator spirometry FEV1 and forced vital capacity (FVC) was measured using KOKO® pneumotachometers (nSpire Health Inc, Longmont, CO) in accordance with American Thoracic Society guidelines [32]. Percent predicted values and lung age were calculated using standard formulas [33]. eCO measurements were performed with portable CO monitor (Breath CO, Clement Clarke Intl., Essex UK) with active smoking defined as an exhaled CO >7 ppm [34]. Self-efficacy and intention to quit smoking was assessed using a modified version of the Prochaska stages of change questionnaire [35]. Nicotine addiction was assessed with the Fagerstrom score [36]. Serum cotinine was measured via radioimmunoassay ELISA (Calbiotech, Spring Valley CA), with a threshold of ≥6 ng/mL indicative of active smoking [37].

### Outcome measure and statistical analysis

The primary outcome of this study was the difference between interventions in six-month biologically-confirmed smoking cessation, defined as self-report of non-smoking in the last seven days combined with negative eCO and serum cotinine at final study visit. Secondary outcomes included differences by intervention in number of visits attended, smoking rates at interim visits (self-report and eCO), number of quit attempts, change in Fagerstrom score, and alterations in self-efficacy. All analyses were based on intention-to-treat. Comparison of outcomes across intervention was performed using chi2 test with Fisher’s exact p-value for small samples of categorical outcomes and t-test or kruskal-wallis for continuous values as appropriate. A p-value of <0.05 was used to define statistical significance. It was determined a priori that if no interaction between lung age and contingency management was observed, individual interventions would be pooled for analysis (i.e., usual care and lung age versus contingency management and contingency management with lung age).

## Results

### Baseline characteristics

**Figure 1:**
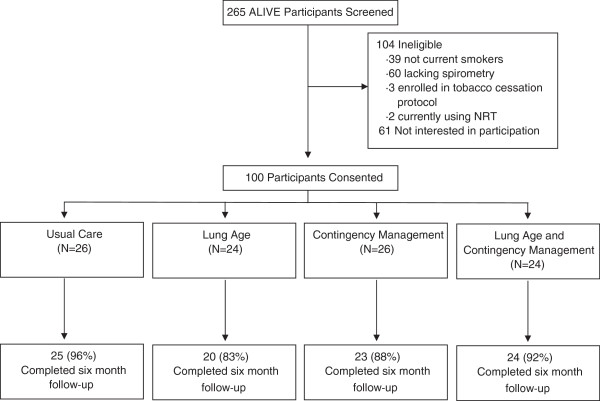
**Study screening, randomization and follow-up.**

A total of 265 ALIVE participants were screened to identify 100 eligible and interested participants for this study (Figure 1). After randomization, 26 participants were enrolled in each of the usual care and contingency management interventions while 24 were enrolled in each of the lung age and lung age combined with contingency management interventions. The median age of the study cohort was 50 (IQR, 45–56) with 47% female participants. The median pack-years smoked was 19 (IQR, 12.5-31), with a median Fagerstrom score of 4 (IQR, 2–5). While all participants had a history of IDU, only 21% reported active injection in the last six months. However, there remained substantial involvement with consumption of other illicit drugs and alcohol, with 32% reporting use of non-intravenous drugs and 51% reporting alcohol use in the last six months. For participants in lung age interventions, the lung age was on average 12 years older than chronological age (median 12; IQR -1 to 23 years older), with 54% of lung age participants having a lung age at least ten years older than chronological age. Randomization resulted in similar baseline characteristics of the four intervention groups (Table 1). There was a higher proportion of non-African Americans in the lung age group when compared to the other interventions.

**Table 1:** **Baseline characteristics at randomization**

	Usual care	Lung age	Contingency management	Lung age + contingency management	p-value
N	26	24	26	24	
Age, years	52.3 (7.2)	46.1 (8.9)	51.2 (7.5)	49.2 (8.7)	0.09
Female, n (%)	11 (42)	10 (42)	17 (65)	9 (38)	0.18
African American race, n (%)	24 (92)	18 (75)	26 (100)	21 (88)	0.03
Current IDU, n (%)	3 (12)	8 (33)	6 (23)	4 (17)	0.27
Current non-IDU, n (%)	7 (27)	8 (33)	7 (27)	10 (42)	0.64
Current alcohol use, n (%)	15 (58)	12 (50)	12 (46)	12 (50)	0.87
More than 1 drink a day per week, n (%)	15 (58)	12 (50)	12 (46)	12 (50)	0.37
Alcohol or drug treatment in last 6 months, n (%)	9 (35)	10 (42)	4 (15)	9 (38)	0.19
HIV infected, n (%)	6 (23)	4 (17)	4 (15)	6 (25)	0.83
Age first smoked	16.2 (5.0)	16.1 (5.4)	17.1 (5.8)	14.9 (4.4)	0.79
Pack-years, med (IQR)	19.1 (13.7-32)	18.4 (14.1-22.8)	17.8 (11.5-31.0)	20.3 (11.0-35.5)	0.74
Smoking >1 pack per day, n (%)	5 (19)	4 (17)	3 (12)	5 (21)	0.68
Smokers in home, n (%)	18 (69)	20 (83)	15 (58)	19 (79)	0.18
Fagerstrom score, med (IQR)	4 (2–5)	3.5 (2–4)	3.5 (2–5)	4 (3–5.5)	0.54
Pulmonary diagnoses					
Asthma, n (%)	6 (23)	5 (21)	8 (31)	5 (22)	0.84
COPD, n (%)	3 (12)	4 (17)	2 (8)	2 (9)	0.79
Both, n (%)	7 (27)	7 (29)	9 (35)	5 (22)	0.79
FEV1					
Absolute (L)	2.58 (0.88)	2.58 (0.78)	2.42 (0.62)	2.72 (0.94)	0.74
% predicted	87.2 (20)	84.1 (19)	86.5 (14)	93.7 (21)	0.42
FVC, (L)					
Absolute (L)	3.58 (1.27)	3.45 (0.96)	3.18 (0.73)	3.62 (1.13)	0.57
% predicted	96.2 (17)	90.8 (14)	91.4 (12)	99.5 (16)	0.14
FEV1/FVC ratio	0.72 (0.11)	0.74 (0.10)	0.76 (0.06)	0.75 (0.09)	0.62
Lung age	--	60.7 (16)	--	54.2 (19.8)	0.31
Difference btw lung age and actual age, median (IQR)	--	12.5 (5.5 to 28.5)	--	11.0 (-10 to 18.5)	0.12

### Outcomes across all interventions

The six month biologically-confirmed smoking cessations rate was 4% for usual care, 0% for lung age, 14% for contingency management and 0% for combined lung age and contingency management. While higher cessation rates were observed in the contingency management intervention, this did not achieve statistical significance (p = 0.13). Using less stringent criteria for smoking cessation (self-report alone or self-report combined with negative eCO) also did not yield differential six month cessation rates across all four interventions. There were no substantial differences in the secondary endpoints of change in smoking rates (self-report and eCO) at interim visits, change in Fagerstrom score, total number of visits attended, alterations in self-efficacy and number of quit attempts comparing the four interventions.

### Outcomes comparing contingency management to non-contingency management

**Figure 2:**
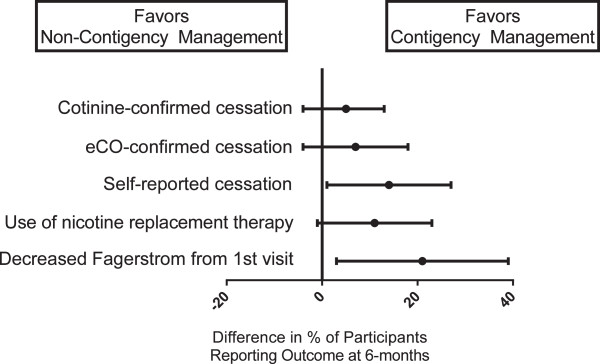
**Relative effect of contingency management on six-month study outcomes.** The figure displays the difference in the percent of participants reporting 6-month outcomes comparing contingency management to non-contingency management interventions. Center points represent the estimated difference in proportions with bars showing 95% confidence intervals. Vertical line represents no difference in proportion between interventions.

**Figure 3:**
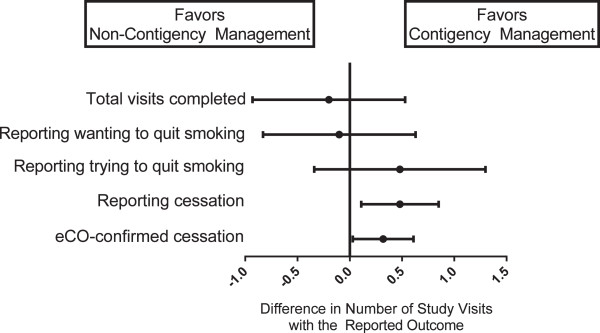
**Relative effect of contingency management on cumulative study outcomes.** Number of Study Visits. The displays the difference in number of cumulative study visits for the stated outcome, comparing contingency management to non-contingency management interventions. Center points represent the estimated mean difference in study visits with bars showing 95% confidence intervals. Vertical line represents no difference in number of visits between interventions.

Because there was neither an effect of lung age on outcomes nor an interaction between contingency management and lung age, we compared outcomes of the contingency management interventions to non-contingency management interventions (usual care combined with lung age alone) (Table 2, Figures 2 and 3). Comparing contingency management interventions (with and without lung age) to non-contingency management interventions (usual care and lung age alone), at six months more individuals self-reported smoking cessation in the prior seven days (18% vs. 4%; p = 0.05). There was no statistically significant difference in biologically-confirmed six month smoking cessation rates between contingency management and non-contingency management interventions (7% vs. 2%; p = 0.36). Contingency management was not associated with higher cumulative study visit attendance (5.14 vs. 5.34 visits; p = 0.59) nor with more visits expressing desire to quit smoking (5.02 vs. 5.12 visits; p = 0.72). However, participants randomized to contingency management interventions had more visits reporting not smoking in the prior seven days confirmed with negative eCO (0.38 vs. 0.06 visits; p = 0.03) and more visits reporting not smoking in the prior seven days (0.58 vs. 0.10 visits; p = 0.01). As well, more participants in the contingency management interventions had a decrease in Fagerstrom score from baseline to six month visit (39% vs. 18%; p = 0.03). The range in decrease of Fagerstrom score was -1 to -2 points for both interventions. There was no difference in measures of self-efficacy between contingency management and non-contingency interventions. Over the study period, in the contingency management interventions a total of 25 participants were reimbursed for an initial negative eCO, 11 for a second negative eCO, four for third negative eCO and one individual achieved four consecutive eCO measurements. The total reimbursement over the six month study period for the 50 eligible participants was $1135 (average of $22.70 per participant).

**Table 2:** **Impact of contingency management on smoking habits and nicotine addiction**

	Non-contingency management	Contingency management	p-value
6 month cotinine confirmed cessation, n (%)	1 (2)	3 (7)	0.36
6 month eCO confirmed cessation, n (%)	2 (4)	5 (11)	0.27
6 month self-reported cessation, n (%)	2 (4)	8 (18)	0.05
Use of nicotine replacement at 6 month visit, n (%)	2 (4)	7 (16)	0.16
Decreased Fagerstrom from 1^st^ visit, n (%)	8 (18)	17 (39)	0.03
Total number of visits^a^, mean (SD)	5.34 (1.83)	5.14 (1.84)	0.59
No. of visits reporting wanting to quit smoking^a^, mean (SD)	5.12 (1.85)	5.02 (1.83)	0.79
No. of visits reporting trying to quit smoking^a^, mean (SD)	1.94 (1.92)	2.42 (2.20)	0.25
No. of visits reporting cessation^a^, mean (SD)	0.10 (0.30)	0.58 (1.28)	0.01
No. of visits with eCO-confirmed cessation^a^, mean (SD)	0.06 (0.24)	0.38 (0.99)	0.03

^a^Number of visits is out of 6 possible.

## Discussion

In this study, we examined the impact of contingency management and the use of spirometric lung age as motivational tools to improve smoking cessation rates among IDUs. While neither intervention, when compared to usual care, was associated with a statistical change in six-month biologically-confirmed cessation rates, we did observe that contingency management interventions were associated with more short-term abstinence as measured by exhaled CO and with lower nicotine addiction as assessed by Fagerstrom score. We did not observe any effect of using spirometric lung age as tool to change smoking behavior or nicotine addiction in this population, and in fact observed that spirometric lung age may have attenuated the effect of contingency management when these interventions were combined.

Contingency management has been reported to enhance smoking cessation rates in opiod-maintained patients [20–22]. Most of these studies were of short duration, and carried out in the context of an existing drug rehabilitation program. Our study extends these findings by testing this intervention outside of a methadone clinic, for a longer duration of follow-up and with rigorous biological confirmation of smoking status. In the setting of a multinational company based in the United States, a longer duration study of more substantial financial incentives ($750 per person over one year) for smoking cessation in a non-substance abusing employee population achieved 15% biologically-confirmed cessation rates at 9-month follow-up compared to 5% in an information-only comparator group [24]. We observed a lower proportion of cessation at the final visit, highlighting the challenges of durable cessation in a substance abusing population. While a statistical difference in 6-month cessation rates was not observed in this study, meaningful differences were observed regarding the beneficial impact of contingency management on outcomes indicative of smoking cessation initiation, suggesting potential efficacy of this intervention in IDUs. These results highlight the potential value in integration of contingency management programs into existing smoking cessation programs for substance abusers.

Contingency management for drug abuse treatment has been extensively evaluated, with consistent data from high-quality studies demonstrating benefit for abuse of varied substances (opiates, stimulants, alcohol, marijuana, tobacco) in different settings (inpatient, outpatient, community-based) [38–44]. Similar to our study, contingencies in studies of drug-users have generally been vouchers or cash-equivalents of modest amount, delivered for providing biological samples which confirm abstinence. Modest incentives ($10) improve attendance at weekly clinic visits among drug users [45] while free methadone vouchers linked 90% of hospitalized drug users to outpatient treatment by 3 months (8 times better than standard referral) [46]. In addition to drug use, modest incentives have been shown to improve participation in HIV counseling and testing, returning to receive HIV test results, and attendance at referral HIV clinic visits [47–49]. Contingency management interventions have demonstrated reductions in HIV-related risk behavior [50, 51], increased compliance with tuberculosis screening [52] and improvements in ART adherence [53–56]. In our population, we have previously observed no difference in smoking behaviors or tobacco-cessation rates by HIV status [28]. Our findings demonstrate that this intervention may be beneficial in changing early smoking habits in a challenging population of current and former drug users, and justify a larger study to explore this intervention further.

While contingency management did impact some outcomes of this study, we did not observe any differential changes in smoking behaviors or perceptions associated with the use of spirometric lung age as a motivational tool. This differs from several prior reports in varied study populations. Parkes and colleagues conducted a randomized controlled trial of smoking cessation incorporating lung age-based counseling in 561 smokers from United Kingdom outpatient practices [57]. At 12 month follow-up, verified quit rates (self-report, carbon monoxide and salivary cotinine) were 6.4% in the control group and 13.6% in the intervention arm (p = 0.005). Average consumption of cigarettes was also lower in the intervention group. The authors conclude that the use of individualized lung age feedback is effective at improving smoking cessation. Lipkus and Prokhorov examined the effect of providing lung age on college smokers’ perceived smoking-related risks, worries and desire to quit [26]. Smokers with a lung age that exceeded their chronological age tended to have greater perceptions of absolute and comparative risk, short- but not long-term worries and expressed a stronger desire to quit. The lung age of this cohort was over a decade older than physiologic age. It is unclear what magnitude of discrepancy between measured and actual age can motivate behavioral changes in this population. While lung age may be beneficial in other populations, the lack of an observed effect in IDUs highlights the need to study specific cessation interventions in unique populations of smokers.

Several theoretical models of health behavior change, including the health belief model, protection motivation theory and precaution adoption models, propose that perceptions of personal vulnerability to the harms of smoking are key to motivating smoking cessation [58–60]. Additionally, greater perceived risk has been associated with a greater desire to quit, more frequent quit attempts and sustained quitting [61–64]. In addition to a lack of impact of lung age as a motivational tool, the benefits of contingency management were not seen when combined with lung age intervention. Based on the null findings observed with lung age-based risk counseling, the relationships between perceived risk and motivation for cessation may be more complex among IDUs. Conceptual models of smoking cessation include reinforcement-based motivational therapy and augmentation of perceived personal risk. Contingency management serves to motivate behavioral change through reinforcement while lung age serves to enhance perceived personal risk. Therefore, the lack of a lung age effect in the population studied here suggests that IDUs may be more responsive to incentives rather than perceived self-risk. Further, the attenuation of contingency management effects by the presence of lung age suggest that lung age may serve as a negative reinforcement of other cessation tools.

Unique strengths of this study include the focus on an under-studied population with high tobacco dependence, a study design permitting evaluation of the independent and synergistic effects of two interventions, and the near-complete 6-month follow-up. This study has some limitations. The small sample size of this study limits the ability to determine the efficacy or estimate the magnitude of the effect of contingency management or spirometric lung age on smoking cessation. The data from this study can be used to inform larger trials testing these interventions. The durability of benefits from contingency management after completion of the intervention is not known, but will be evaluated with on-going follow-up. While the overall rates of cessation were low, a six month 7-11% biological cessation rate is likely substantial in this challenging population of IDUs. Intentionally, this study lacks generalizability outside of urban, minority populations dealing with substance abuse. Our goal was to examine specific cessation interventions among drug users, as this population has high smoking prevalence, substantial tobacco-related disease burden, extremely low cessation rates, and has been greatly understudied in terms of cessation interventions. This study demonstrates that tobacco cessation interventions can be effectively implemented in this population. Novel cessation strategies, such as incorporating mobile health (mHealth) tools to improve smoking cessation among marginalized, underserved populations holds promise for improving access and efficiency to smoking cessation interventions and towards reducing the tobacco-related disparities that currently exist [65].

## Conclusions

In summary, we have demonstrated that contingency management, but not spirometric lung age message, as a motivational tool for smoking cessation leads to more short-term abstinence and lower nicotine addiction over a 6-month period. While definitive benefit on biologically-confirmed smoking cessation at 6 months was not achieved in this study, our findings demonstrate that contingency management interventions are feasible among urban drug-using populations and leads to favorable modifications in smoking behavior, specifically cessation attempts and in reduced levels of nicotine addiction. Larger trials among drug-using populations are appropriate to definitively establish if contingency management is of benefit in this population. Ultimately, contingency management may help decrease the substantial burden of tobacco dependence in similar underserved populations with excessive tobacco use.

## Abbreviations

ALIVE: AIDS Linked to the Intravenous Experience
eCO: Exhaled carbon monoxide
FEV1: Forced expiratory volume in 1 second
FVC: Forced vital capacity
IDUs: Injection drug users.

## References

[1] Marshall MM, McCormack MC, Kirk GD (2009). Effect of cigarette smoking on HIV acquisition, progression, and mortality. AIDS Educ Prev.

[2] Darke S, Hall W (1995). Levels and correlates of polydrug use among heroin users and regular amphetamine users. Drug Alcohol Depend.

[3] Sullivan MA, Covey LS (2002). Current perspectives on smoking cessation among substance abusers. Curr Psychiatry Rep.

[4] Kirk GD, Merlo CA (2011). HIV infection in the etiology of lung cancer: confounding, causality, and consequences. Proc Am Thorac Soc.

[5] Clemmey P, Brooner R, Chutuape MA, Kidorf M, Stitzer M (1997). Smoking habits and attitudes in a methadone maintenance treatment population. Drug Alcohol Depend.

[6] Dell JL, Whitman S, Shah AM, Silva A, Ansell D (2005). Smoking in 6 diverse Chicago communities–a population study. Am J Public Health.

[7] Barbeau EM, Krieger N, Soobader MJ (2004). Working class matters: socioeconomic disadvantage, race/ethnicity, gender, and smoking in NHIS 2000. Am J Public Health.

[8] Honjo K, Tsutsumi A, Kawachi I, Kawakami N (2006). What accounts for the relationship between social class and smoking cessation? Results of a path analysis. Soc Sci Med.

[9] Brook JS, Brook DW, Zhang C (2008). Psychosocial predictors of nicotine dependence in Black and Puerto Rican adults: a longitudinal study. Nicotine Tob Res.

[10] Kanjilal S, Gregg EW, Cheng YJ, Zhang P, Nelson DE, Mensah G, Beckles GL (2006). Socioeconomic status and trends in disparities in 4 major risk factors for cardiovascular disease among US adults, 1971–2002. Arch Intern Med.

[11] Clarke JG, Stein MD, McGarry KA, Gogineni A (2001). Interest in smoking cessation among injection drug users. Am J Addict.

[12] Stark MJ, Campbell BK (1993). Cigarette smoking and methadone dose levels. Am J Drug Alcohol Abuse.

[13] Frosch DL, Shoptaw S, Nahom D, Jarvik ME (2000). Associations between tobacco smoking and illicit drug use among methadone-maintained opiate-dependent individuals. Exp Clin Psychopharmacol.

[14] Higgins ST (2007). Contingency management in substance abuse treatment.

[15] Vlahov D, Strathdee S (2009). The bottom line on cash incentives with drug users. Addiction.

[16] Strong Kinnaman JE, Slade E, Bennett ME, Bellack AS (2007). Examination of contingency payments to dually-diagnosed patients in a multi-faceted behavioral treatment. Addict Behav.

[17] Iguchi MY, Stitzer ML, Bigelow GE, Liebson IA (1988). Contingency management in methadone maintenance: effects of reinforcing and aversive consequences on illicit polydrug use. Drug Alcohol Depend.

[18] Stitzer ML, Iguchi MY, Felch LJ (1992). Contingent take-home incentive: effects on drug use of methadone maintenance patients. J Consult Clin Psychol.

[19] Kidorf M, King VL, Neufeld K, Peirce J, Kolodner K, Brooner RK (2009). Improving substance abuse treatment enrollment in community syringe exchangers. Addiction.

[20] Dunn KE, Sigmon SC, Reimann EF, Badger GJ, Heil SH, Higgins ST (2010). A contingency-management intervention to promote initial smoking cessation among opioid-maintained patients. Exp Clin Psychopharmacol.

[21] Dunn KE, Sigmon SC, Thomas CS, Heil SH, Higgins ST (2008). Voucher-based contingent reinforcement of smoking abstinence among methadone-maintained patients: a pilot study. J Appl Behav Anal.

[22] Alessi SM, Petry NM, Urso J (2008). Contingency management promotes smoking reductions in residential substance abuse patients. J Appl Behav Anal.

[23] Volpp KG, Gurmankin Levy A, Asch DA, Berlin JA, Murphy JJ, Gomez A, Sox H, Zhu J, Lerman C (2006). A randomized controlled trial of financial incentives for smoking cessation. Cancer Epidemiol Biomarkers Prev.

[24] Volpp KG, Troxel AB, Pauly MV, Glick HA, Puig A, Asch DA, Galvin R, Zhu J, Wan F, DeGuzman J, Corbett E, Weiner J, Audrain-McGovern J (2009). A randomized, controlled trial of financial incentives for smoking cessation. N Engl J Med.

[25] Morris JF, Temple W (1985). Spirometric "lung age" estimation for motivating smoking cessation. Prev Med.

[26] Lipkus IM, Prokhorov AV (2007). The effects of providing lung age and respiratory symptoms feedback on community college smokers' perceived smoking-related health risks, worries and desire to quit. Addict Behav.

[27] Vlahov D, Anthony JC, Munoz A, Margolick J, Nelson KE, Celentano DD, Solomon L, Polk BF (1991). The ALIVE study, a longitudinal study of HIV-1 infection in intravenous drug users: description of methods and characteristics of participants. NIDA Res Monogr.

[28] Marshall MM, Kirk GD, Caporaso NE, McCormack MC, Merlo CA, Hague JC, Mehta SH, Engels EA (2011). Tobacco use and nicotine dependence among HIV-infected and uninfected injection drug users. Addict Behav.

[29] Drummond MB, Kirk GD, McCormack MC, Marshall MM, Ricketts EP, Mehta SH, Wise RA, Merlo CA (2010). HIV and COPD: impact of risk behaviors and diseases on quality of life. Qual Life Res.

[30] Vlahov D, Graham N, Hoover D, Flynn C, Bartlett JG, Margolick JB, Lyles CM, Nelson KE, Smith D, Holmberg S, Farzadegan H (1998). Prognostic indicators for AIDS and infectious disease death in HIV-infected injection drug users: plasma viral load and CD4+ cell count. JAMA.

[31] Shoptaw S, Jarvik ME, Ling W, Rawson RA (1996). Contingency management for tobacco smoking in methadone-maintained opiate addicts. Addict Behav.

[32] Miller MR, Hankinson J, Brusasco V, Burgos F, Casaburi R, Coates A, Crapo R, Enright P, van der Grinten CP, Gustafsson P, Jensen R, Johnson DC, MacIntyre N, McKay R, Navajas D, Pedersen OF, Pellegrino R, Viegi G, Wanger J for the ATS/ERS Task Force (2005). Standardisation of spirometry. Eur Respir J.

[33] Hankinson JL, Odencrantz JR, Fedan KB (1999). Spirometric reference values from a sample of the general U.S. population. Am J Respir Crit Care Med.

[34] Deveci SE, Deveci F, Acik Y, Ozan AT (2004). The measurement of exhaled carbon monoxide in healthy smokers and non-smokers. Respir Med.

[35] Prochaska JO, DiClemente CC (1983). Stages and processes of self-change of smoking: toward an integrative model of change. J Consult Clin Psychol.

[36] Heatherton TF, Kozlowski LT, Frecker RC, Fagerstrom KO (1991). The Fagerstrom Test for Nicotine Dependence: a revision of the Fagerstrom Tolerance Questionnaire. Br J Addict.

[37] Wagenknecht LE, Burke GL, Perkins LL, Haley NJ, Friedman GD (1992). Misclassification of smoking status in the CARDIA study: a comparison of self-report with serum cotinine levels. Am J Public Health.

[38] Lussier JP, Heil SH, Mongeon JA, Badger GJ, Higgins ST (2006). A meta-analysis of voucher-based reinforcement therapy for substance use disorders. Addiction.

[39] Stitzer ML, Vandrey R (2008). Contingency management: utility in the treatment of drug abuse disorders. Clin Pharmacol Ther.

[40] Griffith JD, Rowan-Szal GA, Roark RR, Simpson DD (2000). Contingency management in outpatient methadone treatment: a meta-analysis. Drug Alcohol Depend.

[41] Prendergast M, Podus D, Finney J, Greenwell L, Roll J (2006). Contingency management for treatment of substance use disorders: a meta-analysis. Addiction.

[42] Peirce JM, Petry NM, Stitzer ML, Blaine J, Kellogg S, Satterfield F, Schwartz M, Krasnansky J, Pencer E, Silva-Vazquez L, Kirby KC, Royer-Malvestuto C, Roll JM, Cohen A, Copersino ML, Kolodner K, Li R (2006). Effects of lower-cost incentives on stimulant abstinence in methadone maintenance treatment: a National Drug Abuse Treatment Clinical Trials Network study. Arch Gen Psychiatry.

[43] Petry NM, Peirce JM, Stitzer ML, Blaine J, Roll JM, Cohen A, Obert J, Killeen T, Saladin ME, Cowell M, Kirby KC, Sterling R, Royer-Malvestuto C, Hamilton J, Booth RE, Macdonald M, Liebert M, Rader L, Burns R, DiMaria J, Copersino M, Stabile PQ, Kolodner K, Li R (2005). Effect of prize-based incentives on outcomes in stimulant abusers in outpatient psychosocial treatment programs: a national drug abuse treatment clinical trials network study. Arch Gen Psychiatry.

[44] Stitzer M (2006). Contingency management and the addictions. Addiction.

[45] Stitzer ML, Polk T, Bowles S, Kosten T (2010). Drug users' adherence to a 6-month vaccination protocol: effects of motivational incentives. Drug Alcohol Depend.

[46] Sorensen JL, Masson CL, Delucchi K, Sporer K, Barnett PG, Mitsuishi F, Lin C, Song Y, Chen T, Hall SM (2005). Randomized trial of drug abuse treatment-linkage strategies. J Consult Clin Psychol.

[47] Haukoos JS, Witt MD, Coil CJ, Lewis RJ (2005). The effect of financial incentives on adherence with outpatient human immunodeficiency virus testing referrals from the emergency department. Acad Emerg Med.

[48] Thornton R (2008). The Demand for, and Impact of Learning HIV Status. Am Econ Rev.

[49] Rogers RE, Higgins ST, Silverman K, Thomas CS, Badger GJ, Bigelow G, Stitzer M (2008). Abstinence-contingent reinforcement and engagement in non-drug-related activities among illicit drug abusers. Psychol Addict Behav.

[50] Schroeder JR, Epstein DH, Umbricht A, Preston KL (2006). Changes in HIV risk behaviors among patients receiving combined pharmacological and behavioral interventions for heroin and cocaine dependence. Addict Behav.

[51] Shoptaw S, Reback CJ, Peck JA, Yang X, Rotheram-Fuller E, Larkins S, Veniegas RC, Freese TE, Hucks-Ortiz C (2005). Behavioral treatment approaches for methamphetamine dependence and HIV-related sexual risk behaviors among urban gay and bisexual men. Drug Alcohol Depend.

[52] Chaisson RE, Keruly JC, McAvinue S, Gallant JE, Moore RD (1996). Effects of an incentive and education program on return rates for PPD test reading in patients with HIV infection. J Acquir Immune Defic Syndr Hum Retrovirol.

[53] Sorensen JL, Haug NA, Delucchi KL, Gruber V, Kletter E, Batki SL, Tulsky JP, Barnett P, Hall S (2007). Voucher reinforcement improves medication adherence in HIV-positive methadone patients: a randomized trial. Drug Alcohol Depend.

[54] Petry NM, Weinstock J, Alessi SM, Lewis MW, Dieckhaus K (2010). Group-based randomized trial of contingencies for health and abstinence in HIV patients. J Consult Clin Psychol.

[55] Rosen MI, McMahon TJ, Rosenheck R (2007). Does assigning a representative payee reduce substance abuse?. Drug Alcohol Depend.

[56] Simoni JM, Chen WT, Huh D, Fredriksen-Goldsen KI, Pearson C, Zhao H, Shiu CS, Wang X, Zhang F (2010). A Preliminary Randomized Controlled Trial of a Nurse-Delivered Medication Adherence Intervention Among HIV-Positive Outpatients Initiating Antiretroviral Therapy in Beijing, China. AIDS Behav.

[57] Parkes G, Greenhalgh T, Griffin M, Dent R (2008). Effect on smoking quit rate of telling patients their lung age: the Step2quit randomised controlled trial. BMJ.

[58] Janz NK, Becker MH (1984). The Health Belief Model: a decade later. Health Educ Q.

[59] Weinstein ND, Lyon JE, Sandman PM, Cuite CL (1998). Experimental evidence for stages of health behavior change: the precaution adoption process model applied to home radon testing. Health Psychol.

[60] Rogers RW, Petty JTCRE (1983). Cognitive and physiological processes in fear appeals and the attitude change: A revised theory of protection motivation. Social psychosocialphysiology: A sourcebook.

[61] Hansen WB, Collins LM, Johnson CA, Graham JW (1985). Self-initiated smoking cessation among high school students. Addict Behav.

[62] Milam JE, Sussman S, Ritt-Olson A, Dent CW (2000). Perceived invulnerability and cigarette smoking among adolescents. Addict Behav.

[63] Romer D, Jamieson P (2001). Do adolescents appreciate the risks of smoking? Evidence from a national survey. J Adolesc Health.

[64] Rose JS, Chassin L, Presson CC, Sherman SJ (1996). Prospective predictors of quit attempts and smoking cessation in young adults. Health Psychol.

[65] Vidrine DJ, Fletcher FE, Danysh HE, Marani S, Vidrine JI, Cantor SB, Prokhorov AV (2012). A randomized controlled trial to assess the efficacy of an interactive mobile messaging intervention for underserved smokers: Project ACTION. BMC Public Health.

[CR66] The pre-publication history for this paper can be accessed here:http://www.biomedcentral.com/1471-2458/14/761/prepub

